# Modulation of Intracellular ROS and Senescence-Associated Phenotypes of *Xenopus* Oocytes and Eggs by Selective Antioxidants

**DOI:** 10.3390/antiox10071068

**Published:** 2021-07-01

**Authors:** Alexander A. Tokmakov, Ken-Ichi Sato

**Affiliations:** Laboratory of Cell Signaling and Development, Faculty of Life Sciences, Kyoto Sangyo University, Kyoto 603-8555, Japan; kksato@cc.kyoto-su.ac.jp

**Keywords:** oocytes, eggs, aging, *Xenopus laevis*, apocynin

## Abstract

Aging of oocytes and eggs diminishes their reproductive and developmental potential. It has been demonstrated previously that reactive oxygen species (ROS) contribute to accelerated aging of various cells. In the present study, we measured intracellular levels of ROS and investigated effects of several selective antioxidants (AOXs) on the viability and functional activity of aging oocytes and eggs of the African clawed frog *Xenopus laevis*. The fluorescent cell-permeable dye DCFDA, which is widely employed for ROS detection in cultured mammalian cells, was used to monitor ROS levels in the fresh and bench-aged oocytes and eggs by an optimized protocol. It was found that intracellular ROS contents were increased in frog oocytes and eggs aged for 48 h. It was further demonstrated using selective cell-permeable AOXs targeting different ROS-generating mechanisms, that the major source of ROS in *Xenopus* oocytes and eggs is the plasma membrane NADPH oxidase, and that mitochondrial generation contributes to the intracellular ROS content to a lesser extent. Targeted inhibition of NADPH oxidase with a natural organic compound apocynin reduced ROS levels significantly in *Xenopus* oocytes and eggs, maintained their normal phenotype and supported their functional competence. To our knowledge this is the first report concerning beneficial effects of apocynin on the isolated gamete cells, such as oocytes and eggs.

## 1. Introduction

Aging of oocytes and eggs profoundly affects their reproductive and developmental potential. It is thought that the progressive age-associated worsening of oocyte and egg quality represents one of the major causes of infertility and abnormal embryo development in different organisms [1,2,3,4]. Moreover, it was found that postovulatory aging of oocytes also reduces reproductive capacity and longevity of offspring [5,6]. Aging of fully-grown immature oocytes occurs in the ovary during the physiological process called “ovarian aging” [7,8], whereas mature postovulatory oocytes age outside the ovary in the oviduct (in vivo aging) or out-of-body environments (environmental or in vitro aging) [2,9]. 

Both in mammalian and frog species, immature grown-up oocytes reside in the ovaries arrested at the prophase of the first meiotic division. Hormonal stimuli initiate oocyte maturation and ovulation, leading to release of fertilization-competent MII-arrested oocytes from ovarian follicles. These oocytes can be successfully fertilized within several hours following ovulation. In the absence of fertilization, ovulated mammalian oocytes gradually deteriorate in the process of postovulatory aging, undergo spontaneous fragmentation and ultimately die by apoptosis [10,11]. Cytological changes associated with postovulatory aging of mammalian oocytes include partial exocytosis of cortical granules, hardening of the zona pellucida, chromatin disorganization, disruption of the meiotic spindle or its abnormal location, mitochondrial dysfunction, increase in the number of large autophagic lysosomes and apoptotic fragmentation [2,3,9,12]. These changes lead to decreased rates of fertilization, polyspermy, parthenogenesis and abnormal development of embryos. Other established hallmarks of oocyte aging include decrease in maturation-promoting factor (MPF) and mitogen-activated protein kinase (MAPK) activities, disruption of multiple protein kinase signaling pathways, abnormal calcium response and homeostasis, reduced levels of Emi2, decrease in the mitochondrial membrane potential and intracellular ATP content, reduced contents of Bcl-2 mRNA and protein, decrease in glutathione/glutathione disulfide ratio, disruption of lysosome biogenesis, increase in senescense-associated beta-galactosidase (SA-β-gal) activity, multiple epigenetic modifications and elevated levels of reactive oxygen species (ROS) [3,13,14,15,16]. 

At present, the free radical theory of aging is widely accepted to explain deterioration of aging oocytes [13,17,18]. Oxidative stress was demonstrated to impair oocyte quality, cause alterations in the metaphase spindle structure, modulate meiotic cell cycle and induce morphological features characteristic of apoptosis in mammalian oocytes [19,20,21]. Increased intracellular levels of ROS were reported to damage fertilization-initiated calcium signaling and promote age-associated spontaneous activation of mammalian eggs [15,22,23]. Further, extracellular hydrogen peroxide was found to induce intracellular calcium-mediated activation and overactivation of frog eggs [24,25]. It has been hypothesized that oxidative stress may act as the *de facto* initiator of the events that cause aging of postovulatory oocytes [9]. It was proposed that the major mechanism underlying the age-related reduction of oocyte quality is accumulation of mitochondrial damage arising from mitochondria-produced ROS [26]. 

**Figure 1:**
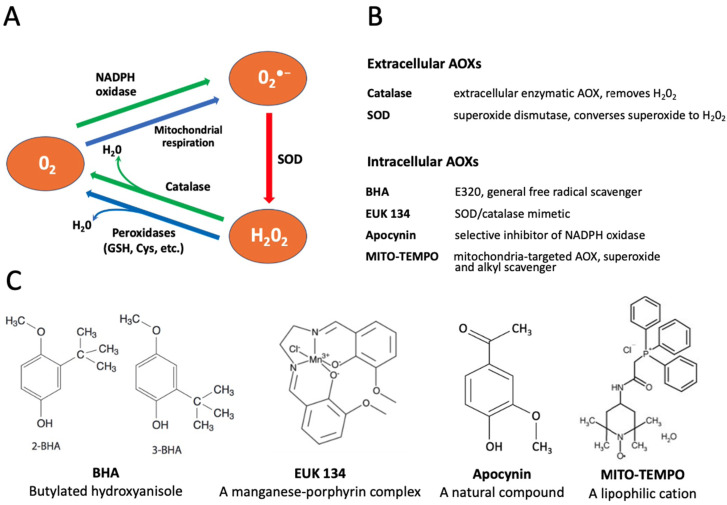
Conversion of reactive oxygen species (ROS) and selectivity of applied antioxidants (AOXs). Panel (**A**) shows the pathways of intracellular conversion of ROS. Panels (**B**,**C**) describe functions and chemical structures, respectively, of the selective AOXs examined in this study.

The reactive oxygen species contribute to accelerated aging of various cells. Generally, the major intracellular sources of superoxide (O_2_^•−^) are the electron transport chain of the mitochondria and the NADPH oxidase system in the cellular plasma membrane [27,28]. Various enzymatic and non-enzymatic agents are employed by cells to detoxify intracellular ROS, including superoxide dismutase (SOD), catalase, peroxidases, glutathione, etc. ([29], Figure 1A). In addition, different antioxidants (AOXs) can be added to culture media to protect oocytes and eggs from oxidative stress.

In the present study, we employed oocytes and eggs of the African clawed frog *Xenopus laevis* to measure intracellular levels of ROS and investigate effects of several selective AOXs on the in vitro aging of these gamete cells. *Xenopus* oocytes and eggs have been widely used in reproductive and cell cycle studies due to their large size, exceeding 1mm in diameter, and superb biochemical tractability. The oocytes and eggs can be obtained abundantly from living female animals and handled in vitro as a primary cell culture. The term “egg” is conventionally used in the frog model for ovulated matured oocytes arrested in the metaphase of the second meiotic division by high activity of cytostatic factor (CSF) and maturation promoting factor (MPF). The meiotic metaphase arrest prevents cell cycle progression and parthenogenesis after meiosis and allows eggs to await fertilization. It has been reported that unfertilized *Xenopus* eggs spontaneously exit the MII arrest and degrade by a well-defined apoptotic process, both in aqueous environments and in the genital tract, within 48 h after ovulation, whereas fully-grown immature *Xenopus* oocytes are remarkably resistant to apoptosis [30,31]. The AOX compounds used in the present study included both the extracellular cell-impermeable agents, such as SOD and catalase, and the intracellular cell-permeable drugs, such as butylated hydroxyanisole (BHA), apocynin, EUK 134 and MITO-TEMPO. The functional targets and molecular structures of these AOXs are detailed in Figure 1B,C.

## 2. Materials and Methods

### 2.1. Reagents

Water-soluble progesterone (PG), anesthetic MS-222 and apocynin were obtained from Sigma (St. Louis, MO, USA). Collagenase (280 U/mg) and catalase (11,000 U/mg) were purchased from Wako (Osaka, Japan), hCG was from Teikoku Zoki (Tokyo, Japan). Fluorogenic caspase-3 substrate IV was purchased from Calbiochem (La Jolla, CA, USA). Hydrogen peroxide colorimetric/fluorometric assay kit was from BioVision (Milpitas, CA, USA). Polyclonal anti-MAPK and anti-pMAPK antibodies were from Cell Signaling (Beverly, MA, USA), biotinylated anti-rabbit IgG was from Vector Laboratories (Burlingame, CA, USA). The Streptavidin Biotin Complex Peroxidase Kit, protein assay CBB solution, SOD (5000 U/mg) and BHA were from Nacalai Tesque (Kyoto, Japan). 2′,7′-dichlorofluorescein diacetate (DCFDA) Cellular ROS Detection Assay Kit was obtained from Abcam (Cambridge, UK), EUK 134 and MITO-TEMPO were ordered from Santa Cruz (Santa Cruz Biotechnology, Dallas, TX, USA). Other chemicals were obtained from Wako and Nacalai Tesque.

### 2.2. Animals and Cells

Adult wild-type female frogs *Xenopus laevis* were purchased from Shimizu (Kyoto, Japan) and maintained in dechlorinated water at the ambient temperature of 21–23 °C. The experiments with the animals were conducted according to the Kyoto Sangyo University Animal Experimentation Regulations under the permission N 2018-20. The experiments with oocytes and eggs were carried out at the ambient temperature of 21–23 °C. To isolate oocytes, the frogs were anesthetized in 2 mg/mL solution of MS-222, then the ovaries were surgically removed and placed into OR-2 solution containing 82.5 mM NaCl, 2.5 mM KCl, 1 mM CaCl_2_, 1 mM MgCl_2_, 1 mM Na_2_HPO_4_, 5 mM HEPES, pH 7.6. The ovaries were manually dissected into clumps of 50–100 oocytes and extensively washed with OR-2 solution. Oocytes were treated with 5 mg/mL collagenase in OR-2 at 21 °C for 3 h by shaking at 60 rpm, extensively washed in OR-2 solution and left for stabilization over 4 h. Undamaged defolliculated oocytes of stage VI were manually selected and used in experiments. The selected cells ranged in size from 1.2 to 1.3 mm. In vitro oocyte maturation was induced by addition of 5 μM PG and monitored by the appearance of a white spot (WS) on the animal hemisphere of oocytes. To obtain eggs for in vitro fertilization experiments, female frogs were injected with 500 IU per animal of hCG and maintained overnight in deionized water complemented with 100 mM NaCl. Ovulation normally began within 10 h after hCG injection. Ovulated or gently squeezed eggs were extensively washed with OR-2 solution and used within 3 h of spawning. Sperm suspension for in vitro fertilization was obtained by macerating one testis surgically removed from an anesthetized frog male in 1 mL of OR-2 and stored on ice until use. Upon fertilization, 0.5 mL of the sperm suspension and 10 mL of 0.2 × OR-2 solution were simultaneously added to the monolayer of eggs placed in a 110-mm-diameter plastic dish. Oocytes and eggs were bench-top aged at the ambient temperature of 21–23 °C in 110 mm dishes for up to 96 h.

### 2.3. Microscopic Observations

Observations of oocyte and egg morphology and cell imaging were carried out using SZX16 stereo zoom microscope (Olympus, Tokyo, Japan) equipped with the high-frame digital microscope CCD camera DP73, CCD interface U-TV0.5XC-3, wide-angle objective SDF PLAPO 1×PF. The CellSens Standard software (Olympus) was used for image acquisition. Acquired images were further processed with the ImageJ software of the National Institute of Health [32].

### 2.4. ROS Detection with DCFDA

**Figure 2:**
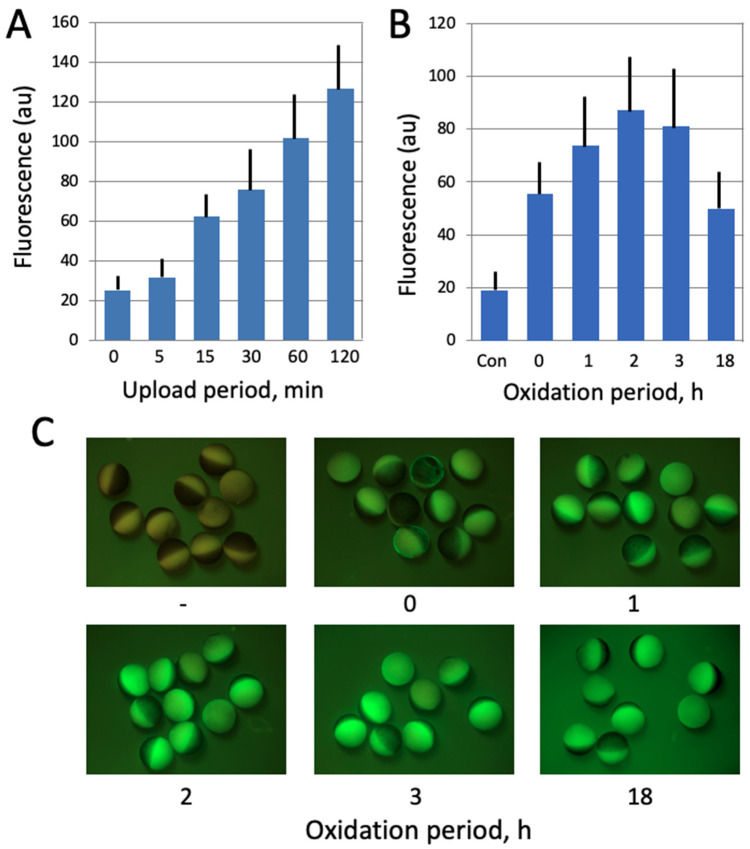
Optimization of ROS detection in *Xenopus* oocytes. In panel (**A**), the oocytes were incubated in the presence of 20 μM DCFDA for the indicated times (0–120 min). At the end of incubation, the indicator was washed off and oocyte fluorescence was observed with the filters used for detection of GFP fluorescence. In panel (**B**), DCFDA was uploaded for 30 min, the drug was washed off, and the oocytes were further incubated in its absence over the oxidation period (0–18 h). Panel (**C**) shows fluorescent images of the oocytes that were analyzed in panel (**B**).

In the present study, a cell-permeant compound 2′,7′-dichlorofluorescein diacetate (DCFDA) was employed to evaluate intracellular ROS contents in *Xenopus* oocytes and eggs. DCFDA is a fluorogenic indicator that measures hydroxyl, peroxyl and other ROS compounds within cells. After diffusion into a cell, the drug is deacetylated by cellular esterases to a non-fluorescent product, which is further oxidized by ROS into a highly fluorescent 2′,7′-dichlorofluorescein (DCF). DCF fluorescence is detected by fluorescent microscopy at 495/529 nm excitation/emission wavelengths. Initially, the protocol for ROS detection with DCFDA was optimized to gain a stronger fluorescent signal. The durations of drug upload and intracellular oxidation were varied, as presented in Figure 2, and the optimal periods of incubation were determined to be 1 h and 2 h for each procedure, respectively. A final concentration of DCFDA in the incubation media was 20 μM. The fluorescent images of oocytes and eggs were taken with the optical interference filters for detection of GFP fluorescence at Ex = 460–495 nm and Em > 510 nm, and the fluorescent signal was quantified using the image acquisition/processing software, as described above in the Section 2.3. The fluorescence from the non-pigmented vegetal hemisphere of oocytes and eggs was quantified.

### 2.5. Treatment of Oocytes and Eggs with AOXs

Defolliculated *Xenopus* oocytes and in vitro matured eggs were incubated in OR-2 at 21 °C with the AOXs used at the following concentrations: catalase and SOD—200 U/mL each, apocynin 0.2—5 mM, as indicated in the figure legends, BHA—100 μM, EUK 134—25 μM, MITO-TEMPO—20 μM. AOX concentrations administered in the present work correspond to those used in previous studies. The incubation times are specified in figure legends.

### 2.6. Immunoblotting

To monitor MAPK phosphorylation levels, supernatant fractions of eggs and oocytes, obtained by centrifugation at 10,000× *g*, 4 °C for 15 min, were incubated at 95 °C for 5 min in the presence of SDS-sample buffer (62.5 mM Tris-HCl, pH 6.8, 2% SDS, 100 mM DTT, 0.01% BPB, 10% sucrose). The samples were subjected to SDS PAGE in 10% polyacrylamide gels and transferred to PVDF membranes using a semidry blotting apparatus (BioRad). The membranes were blocked in T-TBS buffer (20 mM Tris-HCl, pH 7.5, 150 mM NaCl, 0.05% Tween 20) containing 3 mg/mL bovine serum albumin and incubated at RT for 2 h with 100-fold diluted anti-phospho MAPK or 200-fold diluted anti-MAPK antibodies. The membranes were extensively washed with T-TBS buffer and treated with biotinylated anti-rabbit IgG, at a thousand-fold dilution, then with peroxidase-conjugated streptavidin, in accordance with the manual for the Streptavidin Biotin Complex Peroxidase Kit. Color development was catalyzed by the addition of hydrogen peroxide and diaminobenzidine tetra-hydrochloride.

### 2.7. Statistical Analysis

Quantified data in figures are presented as means ± SD values of four to six measurements taken in single batch experiments. All experiments were repeated with the separate batches of oocytes and eggs obtained from at least three different animals. From 50 to 100 oocytes were observed in the experiments that concerned counting oocyte and egg phenotypes, and the signals from 8 to 14 cells were quantified in fluorescent analysis.

### 2.8. Other Methods

Intracellular concentration of hydrogen peroxide was determined using the hydrogen peroxide colorimetric/fluorometric assay kit (BioVision), according to the manufacturer’s manual. Caspase activity assay was performed as described previously [30]. Protein content in the cytosolic fraction of oocytes and eggs was determined with the CBB protein assay.

## 3. Results

### 3.1. Aging of Xenopus Oocytes and Eggs Is Accompanied by ROS Increase 

**Figure 3:**
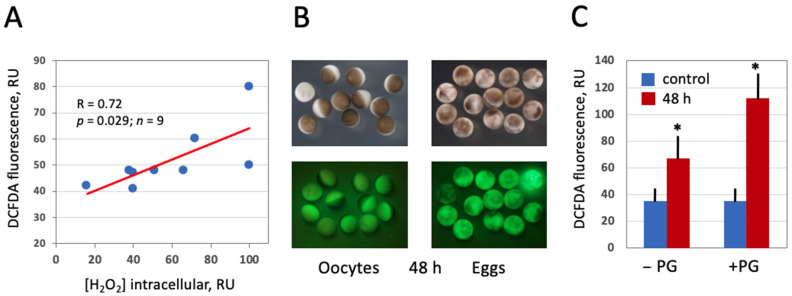
Detection of intracellular ROS in *Xenopus* oocytes and eggs with DCFDA. Oocytes and eggs were preincubated in the presence of 20 μM DCFDA for 1 h followed by incubation in the absence of the dye for 2 more hours. Fluorescence of DCFDA was observed with the filters used for detection of GFP fluorescence. Panel (**A**) demonstrates correlation of DCFDA fluorescence with the intracellular level of hydrogen peroxide. Panel (**B**) shows optical and fluorescent images of oocytes and eggs aged on bench for 48 h, and quantification of DCFDA fluorescence is presented in panel (**C**). Asterisks in panel (**C**) indicate statistical difference from the control (*p* < 0.05).

In this study, the fluorescent cell-permeable indicator DCFDA was used for detection of intracellular ROS in frog oocytes and eggs. The protocol for ROS detection with DCFDA was optimized in the context of this study (Figure 2) to gain a stronger fluorescent signal. The durations of drug upload and intracellular oxidation were determined to be 1 h and 2 h, respectively. Next, we confirmed that the drug can adequately monitor ROS levels in this model. It was found that intensity of the fluorescent signal generated by the indicator correlates significantly with the intracellular content of hydrogen peroxide (Figure 3A), as measured by an alternative method (for details, see Section 2). Then, we investigated whether intracellular ROS contents change during aging of frog oocytes and eggs. Oocytes used in these experiments were surgically removed from the ovaries, defolliculated by collagenase treatment and aged on bench at the ambient temperature. Eggs were obtained by in vitro maturation of the defolliculated oocytes in the presence of progesterone and aged under the same conditions as the oocytes. It was found that intracellular ROS contents were increased in frog oocytes and eggs aged on bench for 48 h (Figure 3B,C). ROS increase in the eggs (i.e., progesterone-treated oocytes) was more prominent than that in the oocytes, however both of the changes were statistically significant (Figure 3C). 

### 3.2. Modulation of Intracellular ROS Levels by Selective AOXs

**Figure 4:**
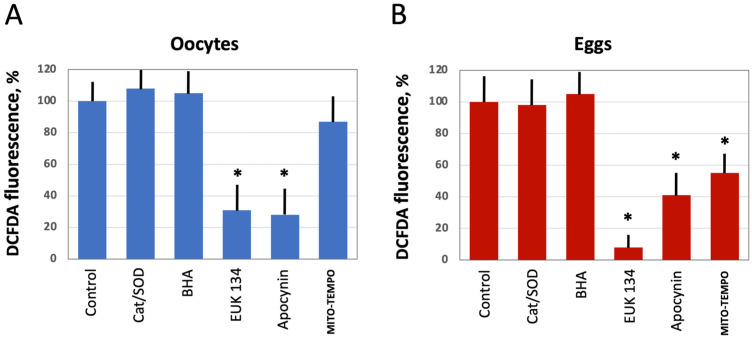
The effect of selective AOXs on ROS levels in oocytes and eggs. Defolliculated *Xenopus* oocytes (**A**) and freshly ovulated dejellied eggs (**B**) were treated with the indicated AOXs, at the concentrations specified in Section 2 “Materials and Methods” for 2 h, then DCFDA fluorescence was detected as described in the legend to Figure 2. Asterisks indicate statistical difference from the control (*p* < 0.05).

Next, we investigated the effect of several selective antioxidants targeting extracellular and intracellular ROS produced by different ROS-generating mechanisms, as detailed in Figure 1. Defolliculated *Xenopus* oocytes and naturally ovulated dejellied eggs were treated with the specified AOXs and loaded with DCFDA, then the indicator-specific fluorescent signal was detected. Markedly, extracellular, i.e., cell-impermeable AOXs, such as catalase (Cat) and superoxide dismutase (SOD), had no effect on intracellular levels of ROS. Moreover, the general cell-permeable free radical scavenger BHA could not affect ROS levels in both oocytes and eggs (Figure 4A,B). However, the cell-permeable SOD/Cat mimetic EUK 134 and selective inhibitor of NADPH oxidase apocynin effectively reduced ROS levels in oocytes and eggs. On the other hand, the mitochondria targeted AOX MITO-TEMPO exerted little effect in oocytes (Figure 4A), but it significantly decreased the fluorescent signal in eggs (Figure 4B). These results indicate that the major source of intracellular ROS in *Xenopus* oocytes and eggs is the plasma membrane NADPH oxidase, and the mitochondrial generation contributes to the intracellular ROS content to a lesser extent.

### 3.3. Modulation of Age-Associated Oocyte Phenotype and Function by Selective AOXs

**Figure 5:**
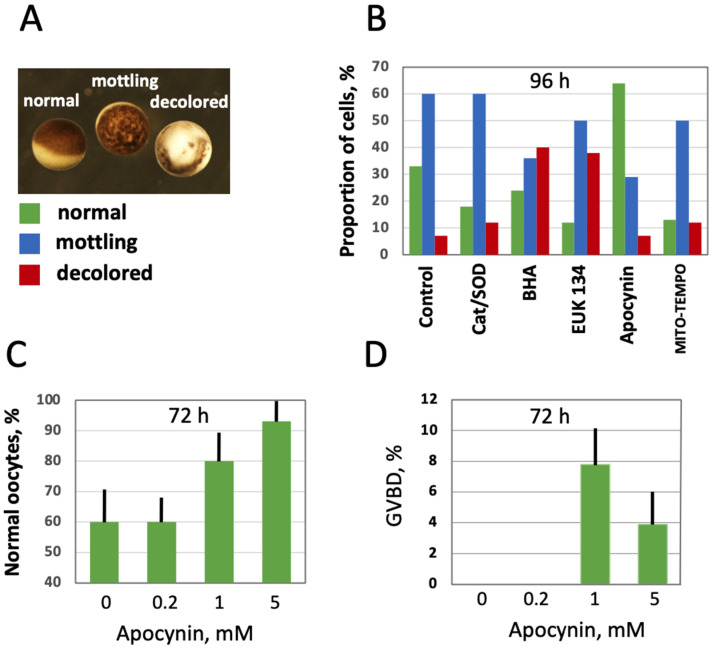
Effect of selective AOXs on the morphology of in vitro aged defolliculated *Xenopus* oocytes. Optical images of normal, mottling and dead oocytes are presented in panel (**A**), proportion of these cell phenotypes in aging oocyte populations treated with the indicated AOXs for 96 h is shown in panel (**B**). Panel (**C**) shows percentage of normal oocytes in the oocyte populations treated with the indicated concentrations of apocynin for 72 h, and panel (**D**) presents occurrence of germinal vesicle breakdown (GVBD) in the aged PG-treated oocytes, as estimated within 12 h of hormone administration. In panel (**D**), apocynin was extensively washed out for 1 h before PG addition to oocytes.

Further, the effect of intracellular ROS level modulation on oocyte morphology and function was investigated in aging populations of oocytes treated with the selective AOXs. The three major categories of oocytes observed in the aging populations were normal, mottling and decolored morphological phenotypes (Figure 5A). Evidently, the mottling and decolored phenotypes are associated with aging as they were not present in the batches of freshly obtained oocytes, and their proportion increased with time. It was found that the proportion of the mottling phenotype was reduced, and the proportion of the normal phenotype was elevated in the oocytes aged on bench for 96 h in the presence of apocynin (Figure 5B). Other cell-permeable AOXs, such as BHA, EUK 134 and MITO-TEMPO, also reduced the content of mottling to some extent, but they also elevated the percentage of decolored oocytes, resulting in decreased proportions of the normal phenotype. As expected, extracellular AOXs, Cat and SOD had little effect on the morphological changes in aging oocytes (Figure 5B). Notably, the effect of apocynin on the morphology of aging oocytes was dose-dependent; it manifested at the drug concentrations exceeding 1 mM (Figure 5C; Supplementary Figure S1). To examine the functional state of oocytes aged in the presence or absence of apocynin, their response to PG was investigated. Markedly, apocynin was noticed to exert an inhibitory effect on oocyte maturation when present in the incubation media (Supplementary Figure S2), so it was extensively washed out one hour before hormone administration. It was found that the oocytes aged on bench for 72 h in the absence of the drug did not respond to PG, whereas some oocytes aged in the presence of apocynin responded to the steroid (Figure 5D). Of note, although the proportion of morphologically normal oocytes was quite high in the oocyte populations aged on bench for 72 h (≥60%), only less than 10% of these cells were maturation-competent (Figure 5D), suggesting that oocyte aging and loss of functional competence can progress in the absence of visible phenotypical changes. A higher rate of in vitro maturation was otherwise reported for *Xenopus* oocytes cultured for 72 h [33], however, the oocytes were incubated at a lower temperature of 18 °C in the previous study. 

### 3.4. Modulation of Age-Associated Egg Phenotype and Function by Selective AOXs

**Figure 6:**
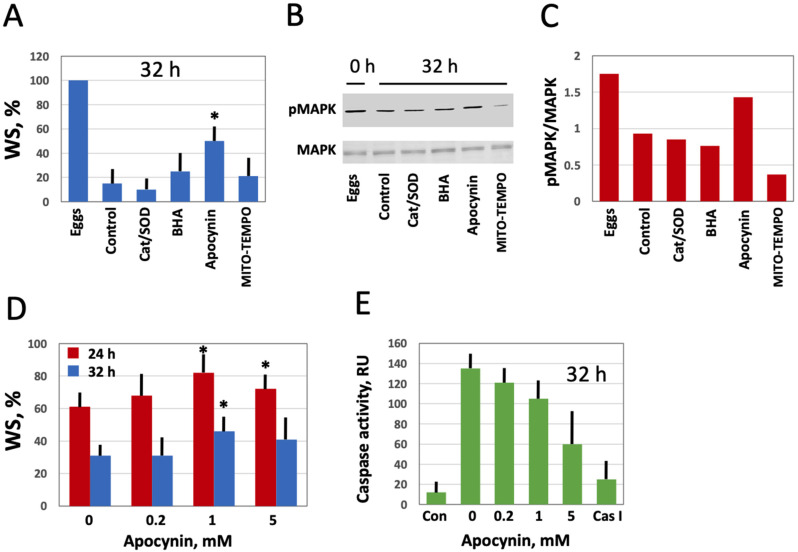
Effect of selective AOXs on the stability of in vitro aged *Xenopus* eggs. Defolliculated oocytes of stage VI were matured in vitro in the presence of PG, then aged on bench in the presence of the specified AOXs. The time after PG administration is indicated in the figure panels. Percentage of the aged eggs bearing a maturation marker, white spot (WS), is presented in panel (**A**). Panels (**B**,**C**) show the activation state of MAPK in the eggs aged in the presence of different AOXs. Proportion of WS-bearing eggs aged in the presence of the indicated apocynin concentrations for 24 and 32 h is evaluated in panel (**D**), and caspase activity in the eggs aged in the presence of various concentrations of apocynin is presented in panel (**E**). The left column in this panel (Con) refers to caspase activity in the eggs matured for 8 to 10 h, and the right column (Cas I) represents caspase activity in the negative control, as measured in the presence of a specific caspase inhibitor Z-VAD-FMK. An asterisk in panel (**A**) indicates statistically significant difference from the control, and asterisks in panel (**D**) denote statistical difference from the egg batch incubated in the absence of apocynin (*p* < 0.05).

**Figure 7:**
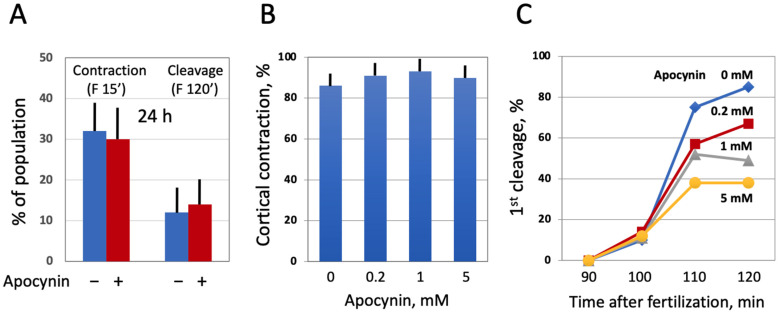
Effect of apocynin on *Xenopus* egg fertilization. In panel (**A**), the eggs obtained from hormone-injected female frogs were aged in the presence or absence of apocynin over 24 h, counting from the time of egg deposition, then the AOX drug was extensively washed off for 1 h, and both treated and untreated eggs were fertilized at the same time. In panels (**B**,**C**), ovulated eggs were fertilized in the presence of apocynin at the indicated concentrations within 1 h after deposition.

Next, we investigated the effect of modulating intracellular ROS on egg morphology and function in aging populations of eggs treated with selective AOXs. It should be noted that *Xenopus* eggs age in vitro much faster than oocytes [30], and the age-dependent increase in the intracellular ROS content is more prominent in the eggs than in the oocytes (Figure 3). It has been reported that the majority of naturally ovulated unfertilized frog eggs spontaneously exit meiotic metaphase arrest and degrade by a well-defined apoptotic process within 48 h of ovulation [30]. Accordingly, it was found that only about 20% of control untreated eggs were able to retain the maturation marker WS by 32 h after PG administration (Figure 6A,D). Apocynin at 1mM increased significantly the WS incidence in the aged eggs by about 50% (Figure 6A,D). Moreover, apocynin-treated eggs displayed an elevated level of MAPK phosphorylation (Figure 6B,C), suggesting that a sizeable fraction of these cells still maintained the meiotic metaphase arrest. Other AOXs tested had little effect on WS occurrence in aged eggs, and phosphorylation level of MAPK was low in the eggs incubated with these compounds (Figure 6A–C). In addition, apocynin inhibited, in a dose-dependent manner, activation of the apoptosis effector caspase, caspase 3/7, that occurs in aging unfertilized *Xenopus* eggs after the meiotic exit (Figure 6E). These data strongly suggested that the proportion of functionally capable fertilization-competent eggs aged in the presence of apocynin would be higher than that in the control egg population untreated with the drug. However, our experiments failed to confirm this suggestion. It was found that the fraction of eggs undergoing cortical contraction and the first embryonic cleavage after fertilization was the same in the apocynin-treated and untreated egg populations aged on a bench for 24 h (Figure 7A). Moreover, it was demonstrated that apocynin, when present during fertilization, exerted a dose-dependent inhibitory effect on furrow formation but not on the cortical contraction (Figure 7B,C). The drug did not delay timing of the first cleavage but rather decreased the proportion of eggs that bore the corresponding phenotype (Figure 7C).

## 4. Discussion

Oxidative stress and intracellular ROS are regarded as the major factors that cause cell and organismal aging. Therefore, attenuation of their adverse effects can potentially slow down the process of aging. Various ROS scavengers and AOXs have been tested with the aim to mitigate oxidative stress in different types of cells and tissues. Several studies have addressed AOX effects on the gamete cells, such as oocytes and eggs. It was reported that supplementing the culture media with AOXs, such as caffeine, vitamin C and E, reduced glutathione or melatonin, was protective against the postovulatory aging of oocytes [9,18,34].

In the present work, we employed frog oocytes and eggs to investigate senescence-associated ROS dynamics and capacity for modulation of cellular phenotypes and functions by several selective AOXs during in vitro aging of these gamete cells. Notably, oocytes and eggs obtained from albino *Xenopus* frogs lacking pigmentation are often used in fluorescent investigations [35,36]. However, our present study utilized the pigmented gamete cells from wild-type animals. This allowed simultaneous detection of intracellular ROS and senescence-specific phenotypic features, such as mottling and decoloring of the animal hemisphere in the aging oocytes, as well as disappearance of WS, a marker of MII arrest, and progressive whitening of the aging eggs.

It was found, using an optimized protocol for intracellular ROS determination based on the detection of DCFDA fluorescence, that both *Xenopus* oocytes and eggs aged on bench for 48 h contain elevated levels of intracellular ROS (Figure 3). This result is consistent with the previous studies reporting an increase in the intracellular ROS content in aging mammalian oocytes and eggs [15,34,37,38]. Of note, increase in ROS contents was more prominent in *Xenopus* eggs than in oocytes. Accordingly, the eggs displayed a typical senescence-specific phenotype that was characterized by the lack of a white spot and decoloring, whereas the oocytes still maintained juvenile morphology after 48 h of aging (Figure 3B). These findings are consistent with our previous observations that unfertilized *Xenopus* eggs spontaneously exit the MII arrest and degrade by an apoptotic process within 48 h after ovulation, whereas fully-grown immature *Xenopus* oocytes are resistant to apoptosis and remain intact for much longer time [30]. Further, it was reported that robust all-out mRNA degradation occurs in apoptotic *Xenopus* eggs, but not in aged oocytes [39]. In connection with our findings, it would be interesting to investigate the content of lipofuscin, a nondegradable complex of oxidized proteins, lipids and metals, in aging *Xenopus* oocytes and eggs. Lipofuscin accumulation is considered as a hallmark of cell senescence and aging [40,41], and elevated levels of intracellular ROS in the aging oocytes and eggs (Figure 2) can be expected to promote lipofuscin accumulation. 

To elucidate the source of ROS in *Xenopus* oocytes and eggs and to alleviate, if possible, adverse effects of intracellular ROS in the aging gamete cells, several selective AOXs were tested in the present study. It was found that the extracellular AOXs, such as SOD and catalase, had no effect on the intracellular levels of ROS in both *Xenopus* oocytes and eggs (Figure 4). Moreover, these AOXs could not mitigate senescence-specific cellular phenotypes observed in the aging cells (Figure 5 and Figure 6). These findings are consistent with the results of a previous study reporting that medium supplementation with catalase and SOD during in vitro maturation and culture did not improve early bovine embryo development [42]. It should be noted, however, that in vitro culture media supplemented with SOD exerted a protective effect from oxidative stress on the development of mouse embryos [43]. Moreover, it was found that diffused intra-oocyte hydrogen peroxide can activate myeloperoxidase and deteriorate oocyte quality [44].

In contrast, cell-permeable AOXs used in this study affected significantly intracellular ROS levels and/or morphological features of *Xenopus* oocytes and eggs. The cell-permeable catalase/SOD mimetic, EUK 134, reduced dramatically intracellular levels of ROS in both *Xenopus* oocytes and eggs (Figure 4), however it was moderately cytotoxic, eliciting the cell death-associated phenotype (Figure 5). Quite surprisingly, the broad range free radical scavenger BHA was ineffective in reducing ROS contents in the oocytes and eggs (Figure 4), however it was cytotoxic too, producing distinctive death-associated phenotypes (Figure 5). The adverse effect of EUK 134 on the oocytes may be related to the fact that certain levels of ROS are indispensable for cell survival, and deep depletion of ROS by this drug might be detrimental to oocyte function. It was reported that administration of broad-range scavengers of oxidative species, such as BHA or *N*-acetyl cysteine, into the ovarian bursa of mice, hormonally induced to ovulate, significantly reduced the rate of ovulation [45]. Evidently, the cytostatic effect of BHA on *Xenopus* oocytes observed in our study is not related to modulation of intracellular ROS, however, it is still unclear why the drug failed to reduce ROS levels in the oocytes and eggs.

Furthermore, our study demonstrates using the selective cell-permeable AOXs, such as apocynin and MITO-TEMPO, that the major source of ROS in *Xenopus* oocytes and eggs is the plasma membrane NADPH oxidase, and that mitochondrial generation also contributes to the intracellular ROS content (Figure 4). Importantly, the inhibitor of NADPH oxidase, apocynin, was found to exert beneficial effects on aging oocytes and eggs (Figure 5 and Figure 6). The drug alleviated, in a dose-dependent manner, senescence-specific phenotypic changes, such as mottling and decoloring of the animal hemisphere in the aging oocytes (Figure 5). It also helped to maintain functional activity of aging oocytes, as it was judged by their boosted ability to respond to progesterone (Figure 5D). In the eggs, apocynin helped to maintain the meiotic metaphase arrest, as revealed by the increased incidence of WS and elevated MAPK phosphorylation in aging populations of eggs incubated with the drug (Figure 6A–D). Moreover, apocynin inhibited dose-dependently caspase activation in aging *Xenopus* eggs (Figure 6E), thus attenuating an apoptotic process that unfolds in the unfertilized frog eggs after meiotic exit [30]. However, our study could not reveal any beneficial effect of apocynin on in vitro fertilization of the aged eggs. Moreover, the drug inhibited furrow formation, when present in media, necessitating its extensive wash before fertilization (Figure 7). Thus, it appears that apocynin can exert both beneficial and adverse effects on the eggs, and it is necessary to thoroughly refine the protocol of drug administration to minimize its adverse influence.

Markedly, apocynin, a natural organic compound isolated from different plants (Figure 1C), has been used previously in various pharmacological studies. Inhibiting NADPH oxidase activity with apocynin was found to exert beneficial effects on skeletal muscle, cardiac and renal fibrosis [46,47,48]. The drug was reported to display anti-inflammatory properties in acute lung inflammation [49,50] and in bronchial asthma [51] where oxidative stress was proven to be involved in disease pathogenesis; apocynin reduced ROS concentrations in exhaled breath condensate in asthmatics. More recently, it was demonstrated that dietary supplementation of apocynin ameliorates an age-related increase in ROS and delays mouse ovarian aging [52]. Now, our current study presents for the first time an evidence for beneficial effects of apocynin on isolated in vitro cultured oocytes and eggs. Identification of the novel AOX compound that can attenuate oxidative stress and delay oocyte aging has the potential to improve fertilization success, especially, in assisted reproduction. Another potential application of promoting oocyte longevity may include advancement of CRISPR-mediated genome editing in *Xenopus* oocytes. A recently developed method based on the use of isolated defolliculated oocytes of *Xenopus laevis* and *Xenopus tropicalis* injected with Cas9 protein/sgRNA reported high-efficiency non-mosaic CRISPR-mediated knock-in and indel mutation in the *Xenopus* model [33]. To achieve a high frequency of mutation using this approach, the injected oocytes were incubated for as long as 72 h, followed by in vitro maturation, thus necessitating delayed oocyte aging and prolonged functional fitness. 

## 5. Conclusions

In sum, our study demonstrates that (i) fluorescent cell-permeable dye DCFDA can be employed for detection of intracellular ROS in isolated *Xenopus* oocytes and eggs; (ii) the major source of ROS in the oocytes and eggs is the plasma membrane NADPH oxidase; (iii) intracellular levels of ROS increase in aging frog oocytes and eggs; (iv) intracellular levels of ROS in *Xenopus* oocytes and eggs can be efficiently modulated by cell-permeable AOXs; (v) apocynin, the selective inhibitor of NADPH oxidase, exerts beneficial effects on the aging oocytes and eggs.

## Data Availability

The data presented in this study are available on request from the corresponding author.

## Supplementary Materials

The following are available online at https://www.mdpi.com/article/10.3390/antiox10071068/s1. Figure S1: Morphology of *Xenopus* oocytes aged in the presence or absence of apocynin. Figure S2: Effect of apocynin on in vitro maturation of *Xenopus* oocytes. Figure S3: Effect of apocynin on in vitro fertilization of *Xenopus* eggs.
