# Supplementary material for: Modulation of Intracellular ROS and Senescence-Associated Phenotypes of Xenopus Oocytes and Eggs by Selective Antioxidants
Source: Antioxidants (Basel). 2021 Jul 1;10(7):1068. doi: 10.3390/antiox10071068 (PMC8301133; doi:10.3390/antiox10071068)
Supplement: Supplementary file 1 [file antioxidants-10-01068-s001.zip › antioxidants-1253565-supplementary.pdf]

antioxidants

MDPI

Article

Modulation of Intracellular ROS and Senescence-Associated Phenotypes of *Xenopus* Oocytes and Eggs by Selective Antioxidants

Supplementary Materials

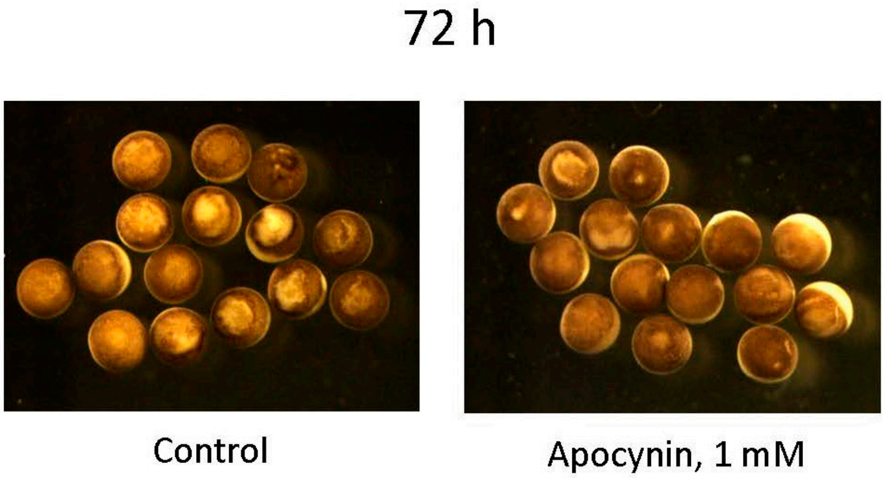

Figure S1. Morphology of *Xenopus* oocytes aged in the presence or absence of apocynin.

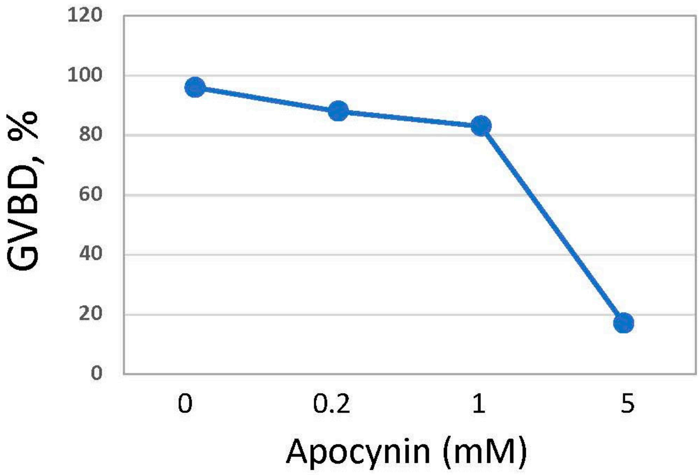

Figure S2. Effect of apocynin on in vitro maturation of *Xenopus* oocytes.

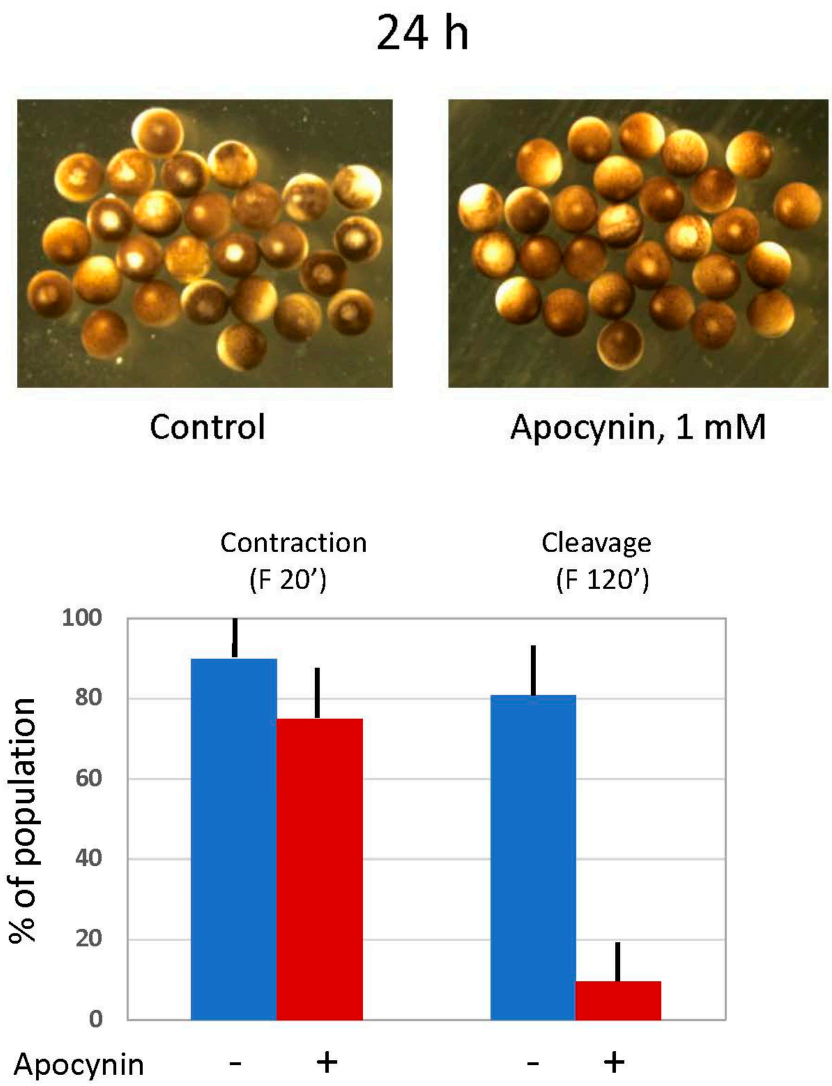

Figure S3. Effect of apocynin on in vitro fertilization of *Xenopus* eggs.
